# Climate Change Impacts on Suitable Habitats of the Endangered *Parnassius imperator*, an Alpine Butterfly Endemic to China

**DOI:** 10.3390/insects17060635

**Published:** 2026-06-16

**Authors:** Keshi Ma, Yongli Wang, Weili Ding, Yiran Ma, Xiaojiao Tang, Jing Han, Junting Li, Xinru Li, Suqin Shang, Mingsheng Yang

**Affiliations:** 1 College of Life Science and Agronomy, and Field Observation and Research Station of Green Agriculture in Dancheng County, Zhoukou Normal University, Zhoukou 466001, China; zknumks@126.com (K.M.); 19337026296@163.com (Y.M.); 18153739652@163.com (X.T.); 19712600197@163.com (J.H.); 15837811493@163.com (J.L.); 19561632707@163.com (X.L.); 2 Biocontrol Engineering Laboratory of Crop Diseases and Pests of Gansu Province, College of Plant Protection, Gansu Agricultural University, Lanzhou 730070, China; 18293673128@163.com; 3 Finance Office, Zhoukou Normal University, Zhoukou 466001, China; dingyi77@163.com

**Keywords:** climate change, habitat suitability, species distribution model, biodiversity conservation

## Abstract

Climate change and habitat loss pose severe threats to the survival of alpine butterflies worldwide. *Parnassius imperator* is a rare, endemic, and endangered butterfly in China, yet the spatiotemporal dynamics of its suitable habitats under climate change remain largely unknown. In this study, we applied ensemble species distribution models to simulate the shifts of its current and future suitable habitats, incorporating bioclimatic variables, elevation, normalized difference vegetation index, and human footprint. Results showed that the current suitable habitats cover 185.87 × 10^4^ km^2^ and are concentrated in western China, mainly regulated by elevation, temperature seasonality (BIO4), precipitation of the wettest month (BIO13), precipitation of the warmest quarter (BIO18), and precipitation of the driest month (BIO14). Under future climate change scenarios, suitable habitats will shrink drastically, even to only 82.16 × 10^4^ km^2^ under SSP585 in the 2070s, with nearly a complete loss of highly suitable habitats. In addition, centroid shift analyses reveal that the distribution centroid will shift eastward. Our findings indicate that suitable habitats will contract significantly, and *P. imperator* will face a sharply increasing risk of extinction in the future. Considering the overlap between suitable habitats and existing nature reserves, we recommend implementing integrated conservation strategies, including expanding protected areas, establishing long-term monitoring programs, restoring habitats, and strengthening law enforcement and public education. This study provides a scientific basis for the climate-adaptive conservation of *P. imperator* and other vulnerable alpine insects.

## Simple Summary

We used ensemble species distribution models combining climate, elevation, vegetation, and human footprint to project current and future habitats for the *Parnassius imperator*, a rare endemic and endangered butterfly in China. Our findings indicate that suitable habitats will contract significantly, and *P. imperator* will face a sharply increasing risk of extinction in the future. We propose expanded protected areas, monitoring, habitat restoration, and public education, providing a scientific basis for climate-adaptive conservation.

## 1. Introduction

Climate change and habitat degradation have exerted profound and far-reaching impacts on the survival and geographical distribution of species worldwide. This often triggers a decline in global biodiversity, which has been formally recognized as the “biodiversity crisis” [1,2]. This ongoing ecological disruption not only undermines the structural stability and functional integrity of entire ecosystems but also poses formidable challenges to global biodiversity conservation efforts [1,3]. Against the backdrop of escalating species loss, especially for threatened and protected taxa, developing science-based strategies to mitigate biodiversity decline has emerged as a pivotal and urgent research focus in the field of ecology [1,3]. Clarifying the spatiotemporal dynamics of species distributions is a fundamental component of ecological and conservation research, as it typically serves as an indispensable prerequisite for formulating and implementing targeted, effective conservation measures [4,5,6].

Species distribution models (SDMs) stand as one of the most prevalent and effective approaches for deciphering species distribution patterns. By correlating the ecological requirements of a target species derived from its known occurrence records with multi-layered environmental predictor datasets, SDMs enable the projection of the species’ potential distribution across diverse habitats that share analogous environmental characteristics [7,8,9]. In practical applications, an ensemble modeling framework, which integrates multiple individual model algorithms (e.g., random forest (RF, Breiman, [10]) and the maximum entropy model (MaxEnt, Phillips et al. [11])), has garnered growing recognition and extensive adoption [8,12,13,14,15,16]. This ensemble approach offers notable advantages over reliance on a single SDM algorithm. For instance, it enhances the robustness and reliability of predictions while alleviating the impacts of inherent biases associated with individual models [17,18,19].

Butterflies are widely recognized as sensitive bioindicators of environmental change, particularly for alpine ecosystems, which are highly vulnerable to climate warming and harbor many rare, endemic, and evolutionarily invaluable species [20,21,22]. The butterfly genus *Parnassius* is recognized as a representative group of alpine butterflies, inherently adapted to high-altitude environments [23]. With ongoing climate warming, these butterflies are forced to shift their habitats toward higher elevations, where suitable montane grassland habitats are increasingly scarce. This habitat compression has rendered several *Parnassius* species highly vulnerable to extinction [4,24]. *Parnassius imperator* Oberthür, 1883, belongs to the genus *Parnassius* of the family Papilionidae in the order Lepidoptera. Among *Parnassius* butterflies, it is a relatively large-sized species and is endemic to China, primarily distributed in Qinghai, Sichuan, Tibet, Yunnan, and Gansu provinces [25,26]. It mainly inhabits alpine meadows, gravel slopes, and valley areas with bare rocks at elevations between 1900 and 3835 m [26,27]. Its larvae feed exclusively on *Corydalis adunca* (Papaveraceae), an endemic herb, and adults rely on specific nectar plants near rocky habitats [26]. Currently, the species faces multiple threats, including climate warming, extreme weather events, habitat loss caused by overgrazing and mining, and human disturbance, all of which have led to population decline and habitat fragmentation [26]. Along with most of *Parnassius* species, *P. imperator* has been listed on the List of Key Protected Wild Animals in China (https://www.forestry.gov.cn/).

In terms of exploring the spatiotemporal dynamics of *Parnassius* distribution via species distribution models (SDMs), a work by Sbaraglia et al. [4] employed the MaxEnt algorithm to simulate the habitat range fluctuations of *Parnassius apollo* throughout the Quaternary glacial cycles. Subsequently, Yu et al. [28] utilized the same algorithm to assess the projected changes in species richness of 59 Papilionidae species (including 11 *Parnassius* taxa) in the Hengduan mountains. More recently, Koo and Park [20] adopted an ensemble modeling approach to evaluate the current and future distribution patterns of *Parnassius bremeri* and its two host plant species in the Republic of Korea. Despite these advances, research on the SDM-based distribution dynamics of *P. imperator* remains notably absent.

In the present study, we constructed ensemble species distribution models under diverse climate change scenarios, integrating key environmental variables including climatic factors, elevation, human footprint (HFP), and the normalized difference vegetation index (NDVI). Our main objectives were: (1) to deepen the understanding of the spatiotemporal distribution dynamics of the ecologically valuable *P. imperator* and the underlying driving factors; (2) to systematically assess the species’ current conservation status and future extinction risk exacerbated by climate change; (3) and ultimately to provide implications for the long-term conservation and sustainable management of *P. imperator* and ecologically related species.

## 2. Materials and Methods

### 2.1. Occurrence Data

**Figure 1:**
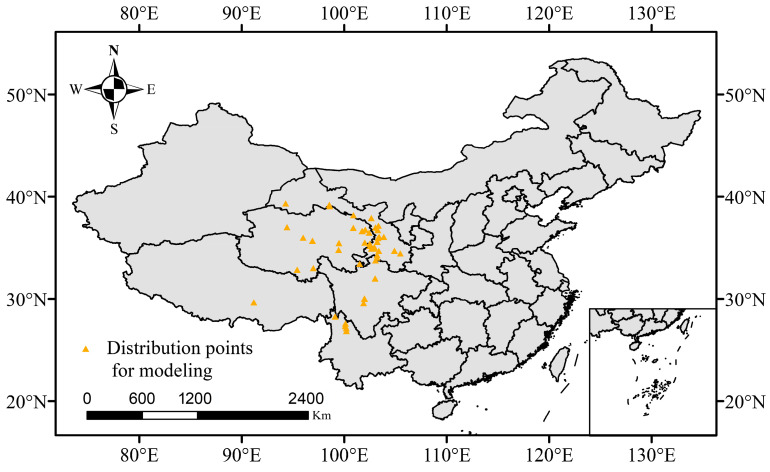
Occurrence records of *Parnassius imperator* in China used in the models.

We collected the distribution records of *P. imperator* mainly from the Global Biodiversity Information website (GBIF, https://www.gbif.org/species/1938659, accessed on 10 March 2024), the published literature searched in the Web of Science (https://www.webofscience.com, accessed 15 March 2024), and the China National Knowledge Infrastructure (https://www.cnki.net, accessed 20 March 2024). The distribution records from the GBIF were acquired using the R package “rgbif” [29] in the form of latitude and longitude coordinates. The distribution information in the literature, which only provided detailed localities such as town names and natural reserve regions, was converted into coordinate forms using the online system (https://api.map.baidu.com/lbsapi/getpoint/, accessed on 10 April 2024). In total, we gathered 63 initial distribution records of *P. imperator*. Then, we used the clean_coordinates function of the R package “CoordinateCleaner” version 2.0 [30] to delete the probable invalid records assigned to the sea, country capitals, or biodiversity institutions. Moreover, to mitigate spatial data bias arising from uneven clustering of occurrence records, such as overconcentration in the same grid cell or easily accessible areas, the dataset was spatially thinned using the R package “spThin” version 0.2.0 [31]. This step ensured no more than one occurrence record per environmental grid cell (approximately 4.5 km^2^). After processing, 48 distribution records were finally retained and used in subsequent analyses (Figure 1, Table S1). We bounded the modeling area with the coordinates (73.42° E, 55.68° N; 136.42° E, 55.68° N; 136.42° E, 18.18° N; 73.42° W, 18.18° N), and all distribution records were within this range.

### 2.2. Variable Selection and Screening

We considered diverse variables associated with climate, topography, normalized difference vegetation index (NDVI), human population density (HPD), and human footprint (HFP) in the modeling to account for the reality that the distribution pattern of one species is jointly determined by various biotic and abiotic factors [32,33]. We obtained 19 bioclimatic variables (BIO1–BIO19) and one topographic variable (elevation) from WorldClim (http://www.worldclim.org/). The climate layers representing the near-current years (1970–2000) and three future periods (2030s: 2021–2040; 2050s: 2041–2060; 2070s: 2061–2080) were downloaded at a 2.5′ spatial resolution. Since different global climate models (GCMs) show different sensitivities to future climate projections, the use of more than one GCM is regarded as improving the reliability of the modeling [5,17]. For each future period, we used three GCMs (BCC-CSM2-MR, IPSL-CM6A-LR, and MRI-ESM2-0) under two shared socioeconomic paths (SSPs: SSP126 and SSP585) in the Coupled Model Intercomparison Project 6 (CMIP6) version [34,35,36,37,38]. Multicollinearity may be present among variables, which can cause model over-fitting. To address this issue, we conducted a Pearson’s correlation analysis among the 19 climatic variables (Figure S1). Then, for the two variables with the correlation coefficient |r| > 0.8 [39,40], the Maximum Entropy (Maxent) algorithm was used to build an initial model to obtain the percentage contribution of each environmental variable, and the one with a lower contribution was removed in subsequent modeling [11,41,42]. Finally, we selected seven bioclimatic variables to predict the potential current and future distribution of *P. imperator* habitats (Table S2). We downloaded the NDVI layers at a spatial resolution of 0.25 km^2^. The remote-sensing vegetation layer data from 2023 (https://lpdaac.usgs.gov/products/mod13q1v061/, accessed on 15 March 2024) were downloaded and further processed using MRT 4.1 (NASA, Washington, DC, USA), ENVI 5.3 (Exelis Visual Information Solutions Corporation, Boulder, CO, USA), and ArcGIS 10.4 (Esri, Redlands, CA, USA), with monthly values aggregated into annual means. In addition, we downloaded the layers of HPD (https://sedac.ciesin.columbia.edu, accessed on 20 April 2024) and HFP (https://www.earthdata.nasa.gov/) at a resolution of 0.25 km^2^. After layer screening, however, we only retained HFP for use in the modeling due to their strong correlation (|r| > 0.8), as also suggested by Li et al. [12]. Finally, all kinds of variable layers were processed with a 2.5′ spatial resolution (with the raster grid about 4.5 km^2^ in size).

Given that elevation strongly influences the distribution of *P. imperator* as an alpine butterfly, and no credible, globally consistent future scenarios are available for NDVI and HFP at the appropriate temporal scales, we constructed models based on two sets of variables: one combining bioclimatic variables and elevation (BIOs + elevation), and the other comprising all variables (BIOs + elevation + NDVI + HFP). The BIOs + elevation model was used for both current and future projections to assess the spatiotemporal dynamics of suitable habitats. The full-variable model was applied to evaluate the relative contributions of all predictors in addition to defining the potential suitable range of *P. imperator* comparable to that under the BIOs + elevation.

### 2.3. Species Distribution Modeling

**Figure 2:**
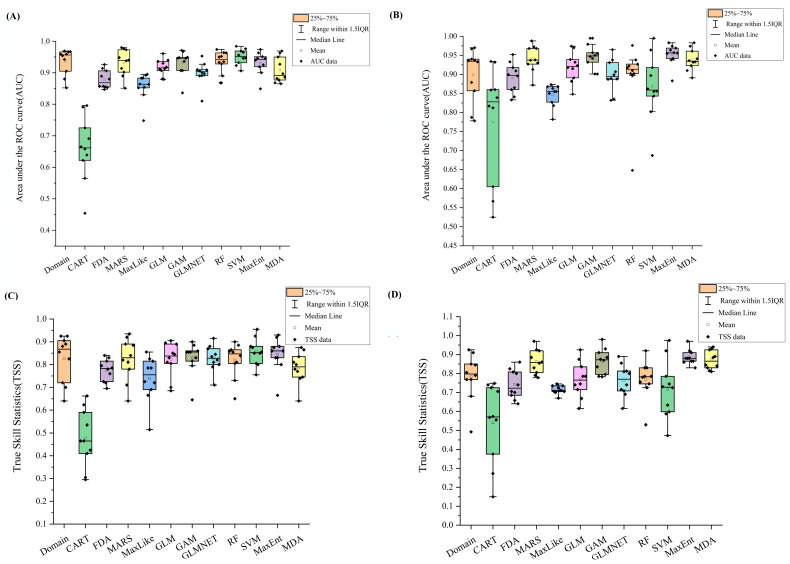
The area under the receiver operating characteristic curve (AUC) and true skill statistics (TSS) values of 12 commonly used species distribution models. (**A**) AUC values under BIOs + elevation; (**B**) AUC values under BIOs + elevation + NDVI + HFP; (**C**) TSS values under BIOs + elevation; (**D**) TSS values under BIOs + elevation + NDVI + HFP.

In this study, ensemble model simulation was implemented in two main steps: individual model selection and ensemble model construction by integrating multiple single models. Both procedures were performed using the R package “sdm” [43]. The detailed modeling workflow is described as follows:

(1) With the occurrence records and environmental layers (BIOs + elevation or BIOs + elevation + NDVI + HFP) as input files, the performance of each of the twelve commonly used model algorithms implemented in the R package “sdm” version 1.1-8 [43] was evaluated. These models included BIOCLIM [44], classification and regression trees (CART) [45], Domain [46], flexible discriminant analysis (FDA) [47], generalized additive model (GAM) [48], generalized linear model (GLM) [49], Glmnet [50], maximum entropy (MaxEnt) [11], Maxlike [51], multivariate adaptive regression spline (MARS) [52], random forests (RF) [10], and support vector machine (SVM) [53].
(2) The main parameters in the evaluation were used as follows. The “gRandom” method of the “sdmData” function was used to randomly generate 1000 pseudo-absences [54]. 75% of the distribution data was set as training data, and the remaining 25% was set as test data. The maximum iterations were set to 5000 [12,55,56]. For each model with a ten-fold cross-validation approach (i.e., 120 single models), the area under a receiver operating characteristic (ROC) curve (AUC) [57] and the true skill statistic (TSS) [58] were calculated. The model with an average AUC ≥ 0.90 and TSS ≥ 0.85 was selected to be used in the following ensemble models (Figure 2).
(3) According to the AUC value ≥ 0.90 and TSS values ≥ 0.85, the top single models (Maxent and SVM for the BIOs + elevation; GAM, MARS, Maxent, and MDA for the BIOs + elevation + NDVI + HFP) were selected for the establishment of ensemble models developed by the R package “sdm” [43].
(4) To construct an ensemble model, the “ensemble” function was used to combine the output results of the selected individual models with a weighted average approach. Besides, the settings of pseudo-absence, division of training and test data, and maximum iterations were the same as those of the selection of single models.
(5) The “getVarImp” function was used to calculate the variable contribution values. Besides, the “roc” and “rcurve” functions were employed to generate the ROC curves for each model and response curves for each variable, respectively. For future projections, the “ensemble” function with a weighted average approach was used as well.

### 2.4. Model Evaluation

We further evaluated the projection performances of two ensemble models using both the average AUC and TSS values. From the modeling, the AUC value generally ranges from 0 to 1. An AUC of 0.7–0.8 is considered acceptable, an AUC of 0.8–0.9 is considered great, and an AUC > 0.9 is considered remarkable [59,60]. In contrast, an AUC value < 0.5 indicates that the performance is no better than random. Due to the equal consideration of sensitivity and specificity in the AUC criterion, which may lead to incorrect evaluation results [61], the TSS value, representing an improved verification index derived from the Kappa coefficient, was considered. This value ranges from −1 to +1. A value of +1 indicates perfect projection, while values of zero or less show that the model performance is no better than random [58,62].

### 2.5. Analyses of Model Results

All predicted habitat suitability values were visualized at the original spatial resolution of 2.5 arc-minutes, consistent with the environmental layers, using ArcGIS 10.4 (Esri, Redlands, CA, USA). Continuous habitat suitability values were derived from individual grid cells (pixels) of the environmental raster datasets. Subsequently, all grid cells were categorized into four suitability ranks, namely, “highly suitable” (0.6–1), “moderately suitable” (0.4–0.6), “lowly suitable” (0.2–0.4), and “unsuitable” (<0.2) [12,16,63]. We used the R package “ggplot2” version 4.0.2 and OriginPro version 2021 (OriginLab Corporation, Northampton, MA, USA) to visualize the response curves showing the effect of each environmental variable on the presence probability of *P. imperator* habitat and the variable importance in the modeling, respectively. To characterize the distribution dynamics between current and future distributions under environmental change, the suitability map was further converted into a binary grid (suitable vs. unsuitable) using a threshold of 0.2, which dichotomized each grid cell into either suitable (>0.2) or unsuitable habitat (<0.2). Accordingly, the maps showing stable, expansion, and contraction regions were generated by comparing the prediction maps under current conditions and climate-change scenarios, and the corresponding areas were calculated with ArcGIS 10.4 (Esri, Redlands, CA, USA). In addition, centroid shift analyses were conducted using ArcGIS 10.4 (Esri, Redlands, CA, USA) to evaluate the core distributional shifts of *P. imperator*, which can visualize the magnitude and direction of the distribution dynamics of *P. imperator* habitat over time.

## 3. Results

### 3.1. Model Performances

**Figure 3:**
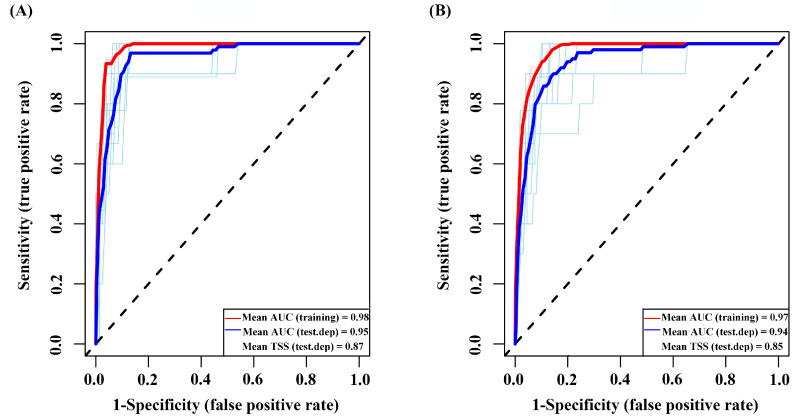
The area under the receiver operating characteristic curve (AUC) and true skill statistics (TSS) values for two used models under BIOs + elevation. (**A**) SVM; (**B**) MaxEnt.

In the ensemble modeling under BIOs + elevation, the mean AUC and TSS values for the two selected models (MaxEnt and SVM) were 0.96 and 0.86, respectively (Figure 3). Under BIOs + elevation + NDVI + HFP, the mean AUC and TSS values for the four selected models (GAM, MARS, MaxEnt, and MDA) were 0.94 and 0.86, respectively (Figure S2). The high AUC and TSS values indicated that the model performances of the two ensemble models were excellent and the predicted habitat suitability was reliable.

### 3.2. Variable Importance

**Figure 4:**
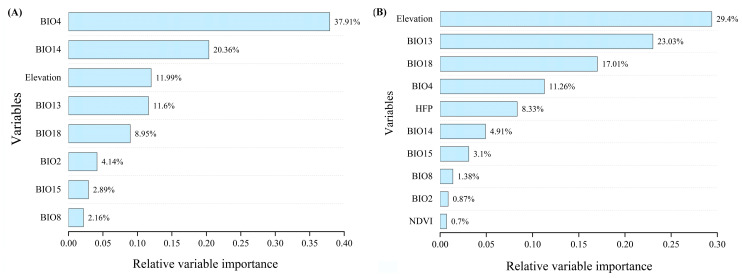
Percent contribution of environmental variables used in the modeling under two variable combinations. (**A**) BIOs; (**B**) BIOs + elevation + NDVI + HFP.

The percentage contributions of each variable to the models are presented in Figure 4. In the model using BIOs + elevation (Figure 4A), BIO4 was the most important predictor (37.91%) for the habitat distribution of *P. imperator*, followed by BIO14 (20.36%), elevation (11.99%), BIO13 (11.6%), and BIO18 (8.95%), while BIO8 made the lowest contribution (2.16%). In the model incorporating BIOs, elevation, NDVI, and HFP (Figure 4B), elevation emerged as the most influential variable (29.4%), followed by the same top three bioclimatic variables identified in Figure 4A: BIO13 (23.03%), BIO18 (17.01%), and BIO4 (11.26%). HFP contributed 8.33%, whereas NDVI had the lowest contribution rate among all variables, at only 0.7%.

### 3.3. Response Curves of the Variables on Presence Probability of P. imperator Habitats

**Figure 5:**
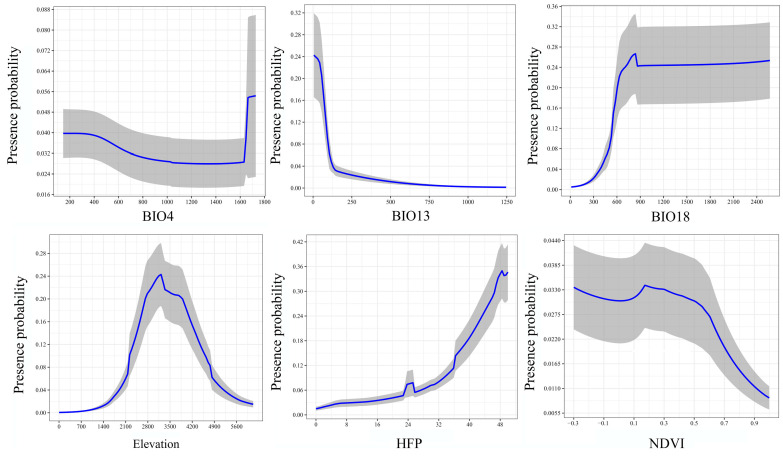
Response curves of the top three bioclimatic variables and nonbioclimatic variables. The blue line indicates the mean predicted presence probability, and the gray shade indicates the confidence interval derived from repeated model runs (cross-validation replicates).

To examine how the probability of *P. imperator* presence varies with environmental variables, response curves for all variables in the BIOs + elevation + NDVI + HFP model are shown in Figure 5. As temperature seasonality (standard deviation×100; BIO4) increased from 200 to 1600, the occurrence probability of *P. imperator* remained relatively stable, ranging from 0.028 to 0.039. For BIO13 (precipitation of the wettest month), the probability peaked at 0.24 at 5 mm, then declined sharply and gradually approached 0 as precipitation increased from 130 to 1250 mm. In contrast, for BIO18 (precipitation of the warmest quarter), the probability rose rapidly from 0 to a maximum of 0.26 at 838 mm, then remained around 0.24 as precipitation increased to 2400 mm. As the top contributing variable, elevation strongly influenced habitat suitability: *P. imperator* favored elevations between 2722 and 3835 m, with occurrence probability >0.2, and the optimal probability (0.243) occurred at 3217 m. Overall, the presence probability increased with HFP values from 0 to 48 but decreased with NDVI values from 0 to 1.

### 3.4. The Current Potential Distribution of P. imperator Habitats

**Figure 6:**
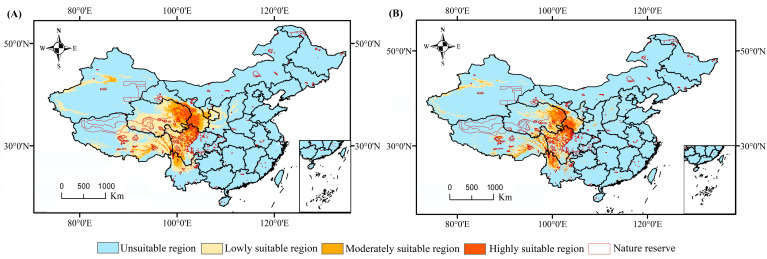
The predicted habitat suitability of *Parnassius imperator* in China under current environmental conditions of two variable combinations. (**A**) BIOs + elevation; (**B**) BIOs + elevation + NDVI + HFP. 0–0.2: unsuitable region, 0.2–0.4: low suitable region, 0.4–0.6: moderately suitable region, 0.6–1: highly suitable region.

Two projections for the current potential distribution of *P. imperator* were conducted based on two variable combinations. Under BIOs + elevation, the suitable habitats (Figure 6A) were distributed in western China, mainly including the provinces of Gansu, Qinghai, Sichuan, Ningxia, Tibet, Shaanxi, Xinjiang, and Yunnan. The total area of suitable habitat was 185.87 × 10^4^ km^2^ (Table S3), with the lowly, moderately, and highly suitable areas being 131.73 × 10^4^ km^2^, 35.5 × 10^4^ km^2^, and 18.64 × 10^4^ km^2^, respectively. The highly suitable regions were primarily distributed in southern Gansu, eastern Qinghai, northwest Sichuan, and southern Ningxia. Under BIOs + elevation + NDVI + HFP, the suitable habitats (Figure 6B) predicted were overall identical to those under BIOs + elevation in distribution patterns of all three levels of suitability. A slight difference was that the suitable regions in Shaanxi, Ningxia, and part of Gansu predicted under BIOs + elevation were evaluated as non-suitable. The current national nature reserves generally cover the core suitable habitats (e.g., northwestern Sichuan, southeastern Qinghai, and northwestern Tibet). However, distinct conservation gaps exist in eastern Tibet, northeastern Qinghai, southern Gansu, and northern Sichuan. The total area of suitable habitats was 118.43 × 10^4^ km^2^, with the lowly, moderately, and highly suitable areas being 70.14 × 10^4^ km^2^, 34.54 × 10^4^ km^2^, and 13.75 × 10^4^ km^2^, respectively.

### 3.5. The Future Potential Distribution of P. imperator Habitats

**Figure 7:**
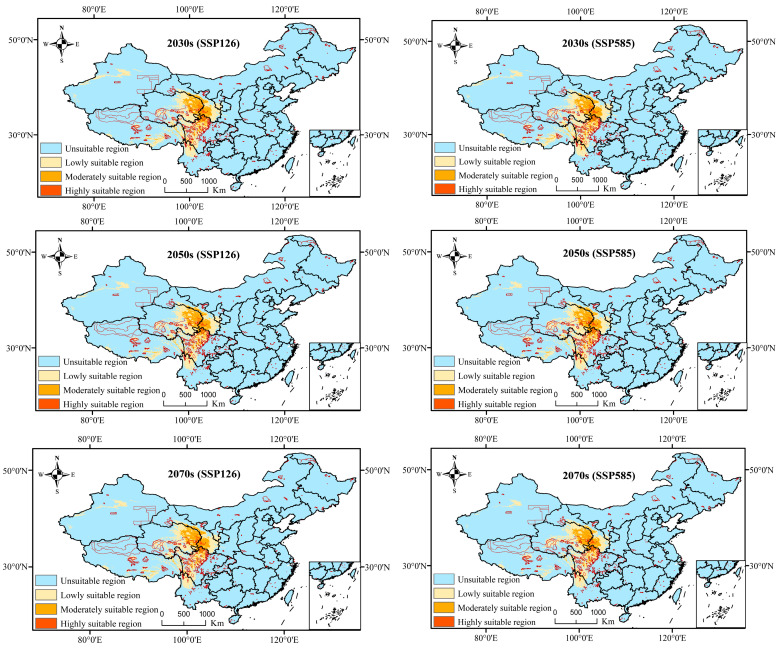
The predicted habitat suitability of *Parnassius imperator* under future scenarios. 0–0.2: unsuitable, 0.2–0.4: low suitable, 0.4–0.6: moderately suitable, 0.6–1: highly suitable. The regions marked with red lines represent national nature reserves.

In future projections under BIOs + elevation, six prediction maps (Figure 7) were derived from 18 predictions based on variables representing three periods, two greenhouse gas emission scenarios, and three global circulation models. Compared with current projections, two notable characteristics emerged. First, the currently suitable habitats significantly contracted in all future scenarios, with an average area of 91.29 × 10^4^ km^2^ (Table S4). The area under SSP585 in the 2070s was even reduced to 82.16 × 10^4^ km^2^. Most of the current habitats that would become lowly suitable or unsuitable for *P. imperator* in the future were mainly distributed in southern Gansu, South Qinghai, West Tibet, and northern Yunnan. Second, most of the suitable habitats were low-suitability habitats defined by a threshold value of 0.2, which occupied 76.6% of the total suitable area on average (Table S4). Moreover, almost no highly suitable habitats were projected across all future climate change scenarios (~0.86 × 10^4^ km^2^). Notably, the current highly suitable habitats in southern Gansu, northeastern Qinghai, and northern Yunnan were projected to disappear entirely. Moreover, these regions lack coverage of national nature reserves.

### 3.6. Change Dynamics of Distribution and Centroid Shift

**Figure 8:**
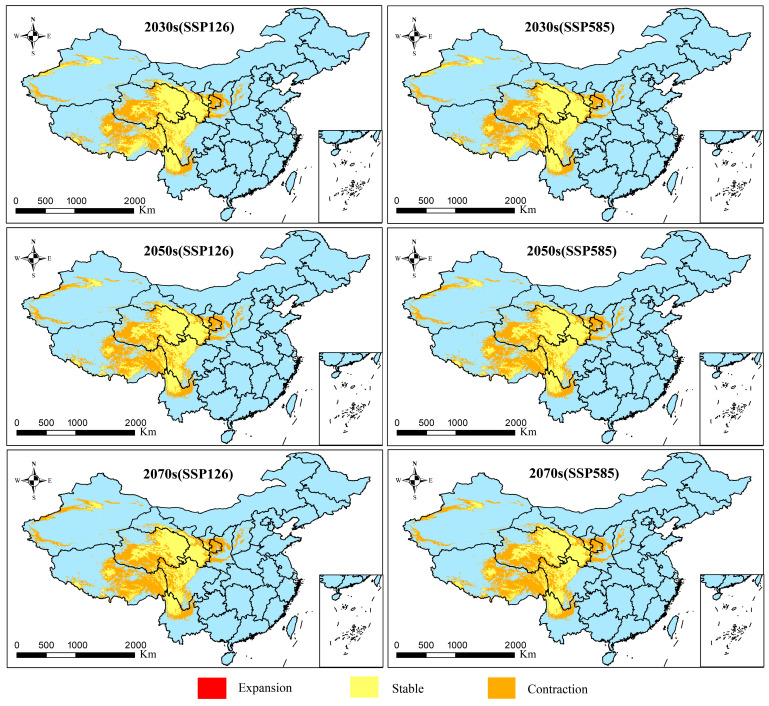
The change dynamics of suitable areas of *Parnassius imperator* under future scenarios relative to that under current conditions.

**Figure 9:**
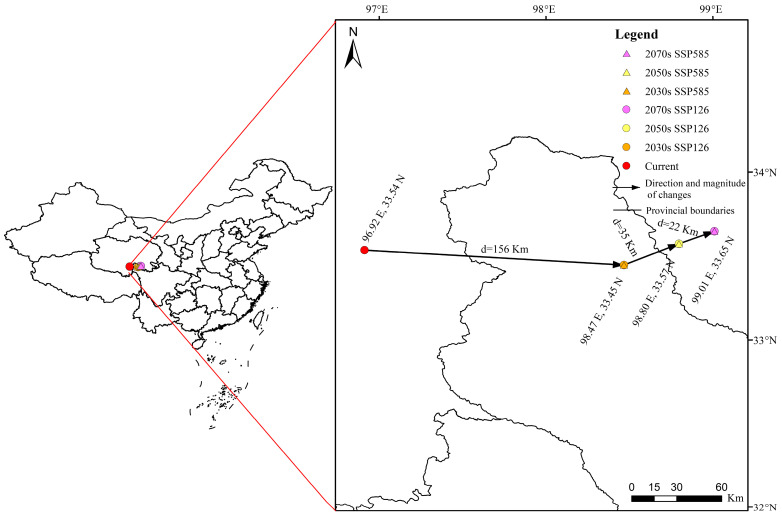
Shifts of distribution cores of *Parnassius imperator* under different scenarios.

The changes in suitable habitat distributions under the future SSP126 and SSP585 scenarios for the 2030s, 2050s, and 2070s relative to the current condition are presented in Figure 8. All future projections indicate a significant contraction of suitable areas, particularly under SSP585 in the 2070s, with only minor expansions observed. The distribution centroids of suitable habitats were predicted to shift within a relatively narrow range (96.92° E–99.01° E, 33.45° N–33.65° N) at the border of Qinghai and Sichuan in southwestern China (Figure 9). Under the current conditions, the habitat centroid was located in Qinghai (96.92° E, 33.54° N) and shifted eastward in all future scenarios. In the 2030s, 2050s, and 2070s, the corresponding shift distances were 156 km, 35 km, and 22 km, respectively.

## 4. Discussion

### 4.1. Suitable Habitats of P. imperator Under Different Climate Scenarios

Under current conditions, the predicted suitable habitats for *P. imperator* are mainly concentrated in western China, including Gansu, Qinghai, Sichuan, Tibet, Yunnan, and Ningxia. This spatial pattern is highly consistent with the field-recorded distribution of *P. imperator* and the biogeographical characteristics of the genus *Parnassius*, which is centered primarily on the Qinghai-Tibet Plateau (QTP) and its surrounding high-altitude mountain ranges [25,26,64,65]. The high consistency between model predictions and actual distributions confirms the reliability of the habitat suitability simulations in this study, complemented by excellent model performance as validated by AUC and TSS metrics [58,60].

Under climate warming, insects often shift their phenology or disperse to higher elevations and latitudes [16,66,67]. Previous SDM-based studies have shown that in response to climate warming, insect species may expand, contract, or stabilize their ranges, reflecting divergent climate responses shaped by habitat characteristics and species-specific adaptations [16,56,68,69]. Under future climate change scenarios, suitable habitats for *P. imperator* will contract significantly across all time periods and emissions pathways. The most severe reduction occurs under the high-emission scenario SSP585 in the 2070s, with suitable area declining to 82.16 × 10^4^ km^2^. Notably, almost no highly suitable habitats are projected under any future scenario, and over 75% of remaining suitable areas are low-suitable habitats, indicating a sharp decline in overall habitat suitability. This pattern aligns with studies of other alpine *Parnassius* butterflies, which report consistent habitat contraction and upward range shifts under warming [4,20,28]. Given the endangered status of *P. imperator*, the substantial contraction of its suitable habitats—especially highly suitable areas—suggests that this butterfly faces elevated risks of local extirpation and potential global extinction. This threat is intensified by increasingly fragmented alpine vegetation and the limited dispersal ability, which impairs its capacity to track suitable environmental conditions [4,24,70].

### 4.2. The Crucial Factors Influencing the Habitat Distribution of P. imperator

Compared with the simulation using climate + elevation variables, the total suitable habitat area predicted by the full-variable model (climate + elevation + NDVI + HFP) is slightly reduced. This indicates that NDVI + HFP also play a vital role in constraining the actual available habitat range of *P. imperator*, alongside climatic drivers. These results are consistent with habitat limitation patterns reported for other alpine *Parnassius* butterflies [4,20].

Among the factors, climate-related temperature and precipitation are widely recognized as pivotal determinants governing species’ geographic ranges [66,69,71]. This linkage arises mainly from their close association with energy and water availability for organisms [72,73]. In our analyses, seven bioclimatic variables were selected: three related to temperature and four to precipitation. Variable importance analyses (Figure 4) revealed that temperature seasonality (BIO4) acted as a dominant climatic driver in the climate + elevation model. For univoltine *Parnassius* species with obligate larval diapause, stable seasonal temperature fluctuations are crucial for synchronizing life cycles with host-plant phenology [23,26,74]. Excessive temperature variability disrupts developmental rates, impairs diapause success, and desynchronizes larvae from their host plants, thereby reducing survival and reproduction. This explains why temperature seasonality strongly constrains the suitable range of *P. imperator*. Precipitation variables, including precipitation of the wettest month (BIO13), precipitation of the driest month (BIO14), and precipitation of the warmest quarter (BIO18), jointly regulated habitat suitability by controlling soil moisture, vegetation productivity, and resource availability in arid alpine ecosystems. Adequate summer moisture supports the growth of *Corydalis* host plants (Papaveraceae), which are essential for larval development, as well as nectar resources for adult butterflies [26,28]. Conversely, excessive rainfall increases infection risk from fungal pathogens and restricts adult flight activity, consistent with physiological limitations in *Parnassius* [20,74].

Elevation is among the most critical environmental drivers, particularly in alpine ecosystems, as it directly shapes both macro- and micro-environmental conditions experienced by species [75,76,77]. In SDM research, elevation has been widely adopted as a key predictor alongside standard bioclimatic covariates [78]. In our modeling, elevation was identified as the primary contributing variable, accounting for 29.4% of the total contribution. This finding indicates that elevation carries greater explanatory power for characterizing the ecological niche of the alpine species *P. imperator* than other environmental variables. This result aligns with the inherent specialization of *Parnassius* butterflies, which are deeply adapted to cool climates, strong ultraviolet radiation, and narrow thermal niches in mountain ecosystems [23,26,28]. Furthermore, the response curves further revealed that *P. imperator* favors habitats at elevations ranging from 2722 to 3835 m, which is highly consistent with field surveys showing that this species mainly inhabits alpine meadows, gravel slopes, and rocky valleys [26]. Such a narrow elevational niche reflects limited dispersal ability and physiological constraints typical of alpine butterflies, which rarely migrate across steep environmental gradients [20,24,70].

In SDM studies, Human population density (HPD) and Human footprint (HFP) have been widely applied as predictors to evaluate the impacts of anthropogenic activities on species distribution or biodiversity [79,80]. For the HPD, most studies assume that rising HPD exacerbates threats to biodiversity [79,81]. However, Luck’s [79] review noted that at broad scales, HPD can be positively correlated with species richness in many spatially congruent taxonomic groups, potentially driven by energy availability. This situation indicates that HPD can exert complex effects on biodiversity [79]. Given the strong correlation between HPD and HFP, we selected HFP as one non-climatic predictor. Our results show that the presence probability increased with HFP values to some extent. Though this trend is unexpected, it aligns with findings from previous insect-related research (e.g., [26,80]). In the study of Li et al. [80], the distribution probabilities of the four grasshoppers increased significantly as the intensity of the human footprint increased. Likewise, abundant flowering plants in areas with human activities can result in higher butterfly diversity [26]. However, we think that this positive correlation observed in our model is limited. *Parnassius imperator* is an endemic alpine butterfly that inhabits high-altitude regions (2722–3835 m, as shown in our response curves; Figure 5). These areas are relatively remote and sparsely populated, resulting in low overall HFP values across the species’ distribution range. In such low-disturbance alpine environments, a positive correlation between HFP and species presence probability may exist to a certain degree.

### 4.3. Implications for P. imperator Conservation

*Parnassius imperator* is a rare butterfly endemic to China and a typical alpine species with high sensitivity to climate change [26]. Our ensemble model results indicate that under future climate scenarios, suitable habitats of *P. imperator* will contract dramatically, almost with a complete loss of highly suitable habitats, suggesting that the species is facing an increasing risk of extinction [24].

*Parnassius imperator* currently receives partial protection from China’s national nature reserves, with core suitable habitats (e.g., northwestern Sichuan, southeastern Qinghai, and northwestern Tibet) largely covered. However, distinct conservation gaps exist in southern Gansu, northeastern Qinghai, and northern Yunnan because these regions support extensive suitable habitats but lack formal protected area designation. Notably, highly suitable habitats in southern Gansu and northern Yunnan are projected to disappear entirely under future climate change. These gaps, combined with climate-driven habitat loss, increase vulnerability to habitat fragmentation and human disturbance [28]. We therefore recommend establishing new protected areas within conservation hotspots, including eastern Tibet, southern Gansu, and northern Yunnan, while also considering the conservation needs of other co-occurring threatened species. Future climate change scenarios should be integrated into long-term conservation planning. Concurrently, priority should be given to safeguarding stable high-altitude habitats as climate refuges, and ex situ conservation measures such as captive breeding should be developed for populations at high extinction risk [4,7].

A standardized long-term monitoring system should be established to track population abundance, distribution, and habitat dynamics of *P. imperator*. Monitoring should focus on marginal low-altitude populations and key habitat contraction zones, providing data to support conservation effectiveness evaluation and adaptive strategy adjustment [28,82]. Degraded alpine meadow habitats within core suitable areas should be restored, and host plants of *P. imperator* should be artificially propagated and protected [20,26]. Finally, it is critical to strengthen law enforcement to suppress illegal collection, and implement public education campaigns to enhance awareness of alpine butterfly conservation and mitigate anthropogenic threats [1,83].

### 4.4. Limitations and Future Prospects

This study explored the habitat dynamics of *P. imperator* using ensemble models, yet it has limitations. We only included climate, elevation, HPD, and NDVI, while ignoring soil, host plants, land-use change, and microclimate, which may also shape the niches of *P. imperator* [4,20]. Furthermore, although we extensively collected the distribution points and the final 48 occurrence records used cover the species’ entire known range, additional field surveys are needed to supplement data from under-sampled regions, which would improve the transferability of future projections [31]. Alternatively, the ensembles of small models can be used to overcome the sampling limitations in modeling rare species [84,85]. In addition, given the high contribution of the elevation factor to the ecological niche of the alpine species *P. imperator*, it could be solely applied to further evaluate its effects on the ecological demand of *P. imperator* and to compare it with other factors. Overall, future research should integrate multi-source environmental variables, expand field occurrence records, or improve methodology to potentially enhance model reliability and support targeted conservation. Additionally, long-term field monitoring of representative *P. imperator* populations should be implemented to validate model projection results, thereby providing robust guidance for the sustainable conservation of *P. imperator* and other *Parnassius* species.

## 5. Conclusions

This study applied ensemble species distribution models to reveal the spatiotemporal dynamics of suitable habitats for *P. imperator*, an endemic alpine butterfly in China. At present, its suitable habitats are mainly concentrated in western China, primarily shaped by elevation, temperature seasonality, precipitation of the wettest month, precipitation of the warmest quarter, and precipitation of the driest month. Under future climate scenarios, suitable habitats will contract sharply, with almost no highly suitable areas projected to persist. Although existing nature reserves cover core habitats, critical conservation gaps remain in regions such as eastern Tibet and southern Gansu. Our findings highlight the increasing extinction risk faced by this species and support the implementation of integrated conservation strategies, including expanding protected areas, establishing long-term monitoring schemes, restoring habitats, and strengthening law enforcement and public education. These results provide a scientific basis for climate-adapted conservation of *P. imperator* and other vulnerable alpine insects.

## Data Availability

The original contributions presented in this study are included in the article/Supplementary Materials. Further inquiries can be directed to the corresponding authors.

## Supplementary Materials

The following supporting information can be downloaded at: https://www.mdpi.com/article/10.3390/insects17060635/s1, Figure S1: Pearson’s correlation analysis among 19 bioclimatic variables; Figure S2: The area under the receiver operating characteristic curve (AUC) and true skill statistics (TSS) values for five used models under BIOs + elevation + NDVI + HFP; Table S1: The occurrence records of *Parnassius imperator* used in the models; Table S2: The environmental variables considered in this study; Table S3: The suitable areas of *Parnassius imperator* under current environmental conditions; Table S4: The suitable areas of *Parnassius imperator* under future scenarios.
