# Supplementary material for: Climate Change Impacts on Suitable Habitats of the Endangered Parnassius imperator, an Alpine Butterfly Endemic to China
Source: Insects. 2026 Jun 16;17(6):635. doi: 10.3390/insects17060635 (PMC13301734; doi:10.3390/insects17060635)
Supplement: Supplementary file 1 [file insects-17-00635-s001.zip › Table S1. The occurrence records of Parnassius imperator used in the models.pdf]

Table S1. The occurrence records of *Parnassius imperator* used in the models

Species	Latitude	Longitude
Parnassius imperator	91.177811	29.65892
Parnassius imperator	94.292439	39.338369
Parnassius imperator	94.422958	37.022648
Parnassius imperator	95.407796	32.854471
Parnassius imperator	96	36
Parnassius imperator	96.914063	35.695096
Parnassius imperator	96.993531	33.012635
Parnassius imperator	98.55085	39.20938
Parnassius imperator	98.598111	39.085861
Parnassius imperator	99.118911	28.28372
Parnassius imperator	99.461278	34.798361
Parnassius imperator	99.4931	35.4689
Parnassius imperator	100.110876	27.352374
Parnassius imperator	100.125527	27.59515
Parnassius imperator	100.210479	27.140138
Parnassius imperator	100.230162	26.861688
Parnassius imperator	100.888458	36.95928
Parnassius imperator	100.90137	38.20748
Parnassius imperator	101.488532	33.434773
Parnassius imperator	101.72	36.623611
Parnassius imperator	101.776109	36.622987
Parnassius imperator	101.878917	29.583917
Parnassius imperator	101.96	30.01
Parnassius imperator	101.96793	30.05426
Parnassius imperator	101.99138	35.526274
Parnassius imperator	102.05	36.766667
Parnassius imperator	102.413889	36.47924
Parnassius imperator	102.492951	35.41046
Parnassius imperator	102.516667	35.2
Parnassius imperator	102.638194	37.928278
Parnassius imperator	102.649307	34.935116
Parnassius imperator	102.900598	34.938586
Parnassius imperator	102.922631	35.093868
Parnassius imperator	103	32
Parnassius imperator	103.032289	36.988522
Parnassius imperator	103.036871	33.769759
Parnassius imperator	103.191848	34.055184
Parnassius imperator	103.207	34.302
Parnassius imperator	103.218501	35.608021
Parnassius imperator	103.265473	36.742096
Parnassius imperator	103.292503	35.963331

Parnassius imperator	103.333333	37.166667
Parnassius imperator	103.360532	34.699114
Parnassius imperator	103.388934	36.080457
Parnassius imperator	103.845123	36.065368
Parnassius imperator	104.89685	34.72767
Parnassius imperator	105.46	34.45556
Parnassius imperator	110.187088	20.010042
