# Supplementary material for: Climate Change Impacts on Suitable Habitats of the Endangered Parnassius imperator, an Alpine Butterfly Endemic to China
Source: Insects. 2026 Jun 16;17(6):635. doi: 10.3390/insects17060635 (PMC13301734; doi:10.3390/insects17060635)
Supplement: Supplementary file 1 [file insects-17-00635-s001.zip › Table S2. The environmental variables considered in this study.pdf]

Table S2. The environmental variables considered in this study

Variable type	Variable	Description
Bioclimatic Variables	BIO1	Annual mean temperature (°C)
	**BIO2**	**Mean diurnal range (mean of monthly (max temp-min temp)) (°C)**
	BIO3	Isothermality (bio2/bio7) (× 100)
	**BIO4**	**Temperature seasonality (standard deviation ×100)**
	BIO5	Max temperature of warmest month (°C)
	BIO6	Min temperature of coldest month (°C)
	BIO7	Annual temperature range (bio5–bio6) (°C)
	**BIO8**	**Mean temperature of wettest quarter (°C)**
	BIO9	Mean temperature of driest quarter (°C)
	BIO10	Mean temperature of warmest quarter (°C)
	BIO11	Mean temperature of coldest quarter (°C)
	BIO12	Annual precipitation (mm)
	**BIO13**	**Precipitation of wettest month (mm)**
	**BIO14**	**Precipitation of driest month (mm)**
	**BIO15**	**Precipitation seasonality (coefficient of variation)**
	BIO16	Precipitation of wettest quarter (mm)
	BIO17	Precipitation of driest quarter (mm)
	**BIO18**	**Precipitation of warmest quarter (mm)**
	BIO19	Precipitation of coldest quarter (mm)
Topography	**Elevation**	**Ground height above sea level (m)**
Vegetation	**NDVI**	**Normalized difference vegetation index**
Human activity	HPD	Human population density
	**HFP**	**Human footprint index**

Note: the variables in bold were finally used in the modeling.
