# Supplementary material for: Climate Change Impacts on Suitable Habitats of the Endangered Parnassius imperator, an Alpine Butterfly Endemic to China
Source: Insects. 2026 Jun 16;17(6):635. doi: 10.3390/insects17060635 (PMC13301734; doi:10.3390/insects17060635)
Supplement: Supplementary file 1 [file insects-17-00635-s001.zip › Table S3. The suitable areas of Parnassius imperator under current environmental conditions.pdf]

Table S3. The suitable areas ($\times 10^4$ km^2) of *Parnassius imperator* under current environmental conditions. The percentages in parentheses indicate the proportion of suitable areas occupied by the land area of China.

**Areas**	**Low suitability (0.2–0.4)**	**Moderate suitability (0.4–0.6)**	**High suitability (0.6–1)**	**Suitable habitats (0.2-1)**
BIOs + elevation	131.73 (13.72%)	35.5 (3.70%)	18.64 (1.94%)	185.87 (19.36%)
BIOs + elevation + NDVI + HFP	70.14 (7.31%)	34.54 (3.60%)	13.75 (1.43%)	118.43 (12.34%)
