# Supplementary material for: Climate Change Impacts on Suitable Habitats of the Endangered Parnassius imperator, an Alpine Butterfly Endemic to China
Source: Insects. 2026 Jun 16;17(6):635. doi: 10.3390/insects17060635 (PMC13301734; doi:10.3390/insects17060635)
Supplement: Supplementary file 1 [file insects-17-00635-s001.zip › Table S4. The suitable areas of Parnassius imperator under future scenarios.pdf]

Table S4. The suitable areas (× 10^4 km^2) of *Parnassius imperator* under future scenarios. The percentages in parentheses indicate the proportion of suitable areas occupied by the land area of China.

Climate conditions	**Low suitability (0.2–0.4)**	**Moderate suitability (0.4–0.6)**	**High suitability (0.6–1)**	**Suitable habitats (0.2-1)**
current	131.73 (13.72%)	35.5 (3.70%)	18.64 (1.94%)	185.87 (19.36%)
2030s SSP126	73.72 (7.68%)	21.65 (2.26%)	0.86 (0.09%)	96.23 (10.02%)
2030s SSP585	76.73 (7.99%)	22.25 (2.32%)	0.85 (0.09%)	99.83 (10.40%)
2050s SSP126	68.41 (7.13%)	20.07 (2.09%)	0.82 (0.09%)	89.30 (9.30%)
2050s SSP585	68.79 (7.17%)	21.12 (2.20%)	0.90 (0.09%)	90.81 (9.46%)
2070s SSP126	68.44 (7.13%)	20.03 (2.09%)	0.94 (0.09%)	89.41 (9.31%)
2070s SSP585	63.49 (6.61%)	17.89 (1.86%)	0.78 (0.08%)	82.16 (8.56%)
