# Supplementary figures and images for: Climate Change Impacts on Suitable Habitats of the Endangered Parnassius imperator, an Alpine Butterfly Endemic to China
Source: Insects. 2026 Jun 16;17(6):635. doi: 10.3390/insects17060635 (PMC13301734; doi:10.3390/insects17060635)

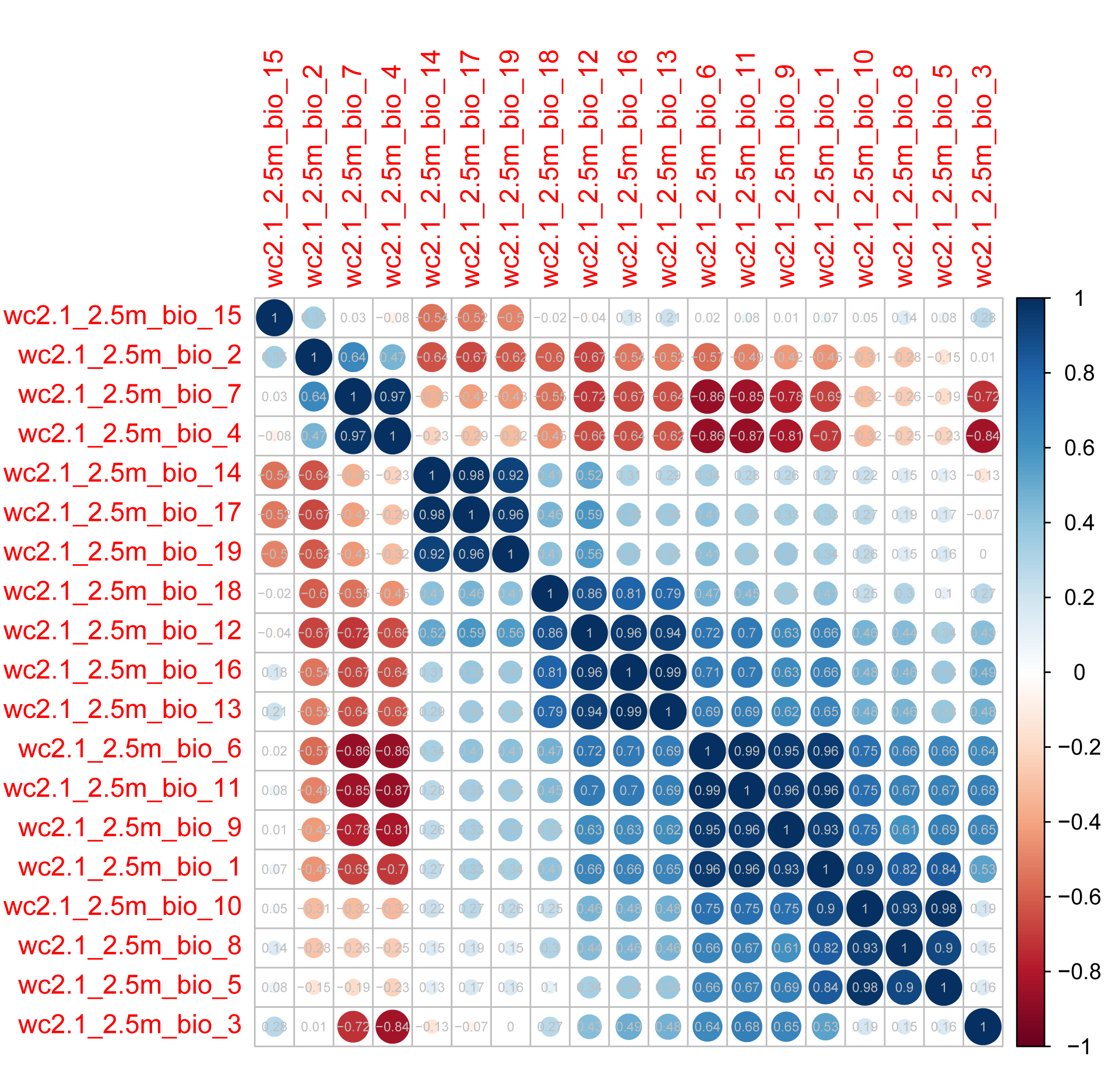

Supplement: Supplementary file 1 [file insects-17-00635-s001.zip › Figure S1.tif]

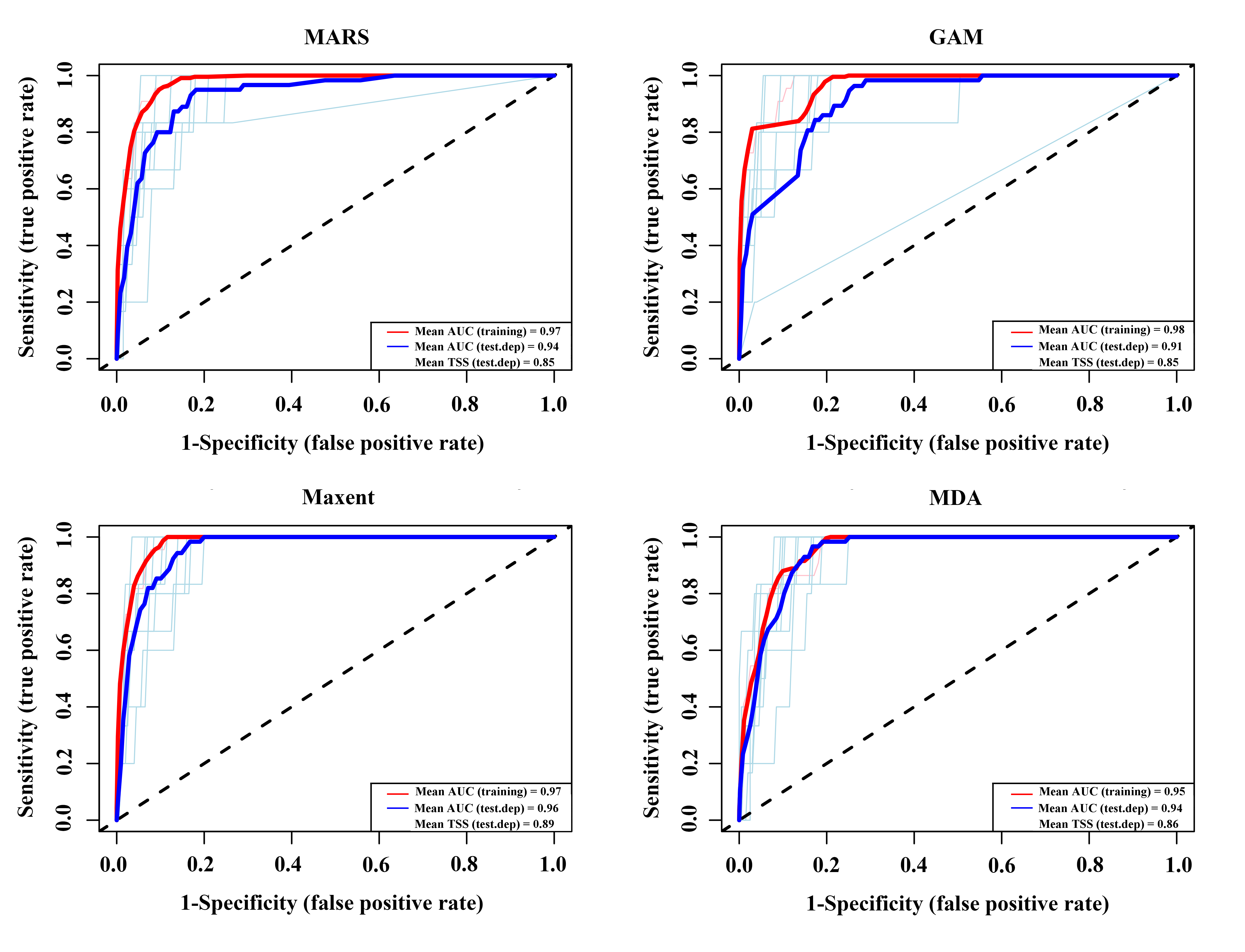

Supplement: Supplementary file 1 [file insects-17-00635-s001.zip › Figure S2.tif]
